# Structure–property relations of a unique and systematic dataset of 19 isostructural multicomponent apremilast forms

**DOI:** 10.1107/S2052252522005577

**Published:** 2022-06-15

**Authors:** Jan Jirát, Martin Babor, Luděk Ridvan, Eliška Skořepová, Michal Dušek, Miroslav Šoóš

**Affiliations:** a Department of Chemical Engineering, University of Chemistry and Technology, Technická 3, Prague 6 16628, Czech Republic; b Zentiva, k.s, U kabelovny 130, Prague 10 10237, Czech Republic; c Department of Solid State Chemistry, University of Chemistry and Technology, Technická 5, Prague 6 16628, Czech Republic; d Institute of Physics of the Czech Academy of Sciences, Na Slovance 2, Prague 8 18200, Czech Republic

**Keywords:** apremilast, structure–property relations, multicomponent forms, equilibrium solubility, bioavailability

## Abstract

The structure–property relations are examined for apremilast cocrystals and solvates in this work. A unique and large dataset of multicomponent crystal forms is presented including 7 cocrystals and 12 solvates. In total, 15 of the presented multicomponent forms and their crystal structures are published here for the first time. This dataset is unique owing to the extreme crystal packing similarity of all 19 crystal forms. This fact makes the evaluation of structure–property relations significantly easier and more precise since the differences in the crystal lattice arrangement are close to negligible. Properties of the guest molecules used here can be directly correlated with the macroscopic properties of the corresponding multicomponent forms. Interestingly, a considerable correlation was found between the intrinsic dissolution rate of the multicomponent forms and their solubility, as well as the solubility of their guest molecules in the dissolution medium. The latter is of particular interest as it can aid in the design of multicomponent forms with tuned properties.

## Abstract 2

A unique system of 19 isostructural apremilast multicomponent forms was explored and a correlation between the intrinsic dissolution rates of the new solid forms and the equilibrium solubility of their guest molecules has been discovered.

## 1. Introduction

In recent years, the pharmaceutical industry has been challenged with the task of increasing the solubility of poorly water-soluble drug products (Good & Rodríguez-Hornedo, 2009 ▸; Blagden *et al.*, 2007 ▸; Chen *et al.*, 2011 ▸). The biggest problem for oral drug administration – the most common method of treatment – is low bioavailability (Gavhane & Yadav, 2012 ▸; Pinnamaneni *et al.*, 2002 ▸), which is a consequence of poor aqueous solubility (Lipinski, 2000 ▸). This issue is expected to be increasingly relevant in the coming years. In the current market, approximately 40% (Lipinski, 2002 ▸) of active pharmaceutical ingredients (APIs) have poor solubility and this number is expected to increase to 70–90% in the future (Thayer, 2010 ▸). Ongoing advances motivate academic and industrial researchers in the field of crystal engineering to search for modern multicomponent solid-state forms, *i.e.* salts, solvates and cocrystals. The formation of a new multicomponent form can significantly increase not only the solubility and thus the bioavailability, but also improve other important properties such as thermal stability, hygroscopicity, powder flowability, and physical as well as chemical stability (Qiao *et al.*, 2011 ▸; Jia *et al.*, 2015 ▸; Stanton & Bak, 2008 ▸). These properties are crucial in the process of drug development. However, designing new multicomponent forms with desired properties remains a difficult task. It requires skilled researchers and significant experimental and cost efforts, since a comprehensive theory describing the formation of such solids is yet to be developed. Further, the links between solid-state structures and macroscopic properties, such as solubility or thermal stability, are generally not well explored or understood. Thus, contributions from experimental research as well as mathematical modelling are essential to deepeen our understanding of the formation of new multicomponent forms and the relation between their crystal structure and macroscopic properties. Each contribution provides knowledge that helps to minimize experimental efforts for researchers and reduce industrial expense to make medicines more affordable.

The connection between the multicomponent crystal form and its properties has mostly been explored within salts (de Moraes *et al.*, 2017 ▸; Arlin *et al.*, 2011 ▸; Collier *et al.*, 2006 ▸; Black *et al.*, 2007 ▸). However, many proposed structure–property relations are valid only for small systems and cannot necessarily be applied to broader systems or different compounds. For example, the lack of versatility can be illustrated by contradicting observations, *i.e.* adding polar groups to API species can result in both increased as well as decreased aqueous solubility (Agharkar *et al.*, 1976 ▸; Parshad *et al.*, 2004 ▸). This problem is exacerbated by the lack of larger, systematically explored datasets that could provide both structural information of crystal forms as well as evaluation of reliable physicochemical data of specific forms, which is crucial for a clearer understanding of the structure–property relation. However, previous studies with larger sample sizes did not contain structural data (Streng *et al.*, 1984 ▸). Recent studies report both the crystal structures and the properties of the prepared salts. Unfortunately, most of them contain a relatively small number of samples (Sanphui *et al.*, 2014 ▸; Mannava *et al.*, 2020 ▸; Gunnam & Nangia, 2019 ▸; Banik *et al.*, 2016 ▸; Martin *et al.*, 2013 ▸; Thakuria & Nangia, 2013 ▸; Goud *et al.*, 2013 ▸). Several studies with systematic large datasets are now available (20–50 salts) that bring new insight into this topic (de Moraes *et al.*, 2017 ▸; Arlin *et al.*, 2011 ▸; Collier *et al.*, 2006 ▸; Black *et al.*, 2007 ▸). These systematic studies currently available for salts are not yet available for cocrystals and solvates, despite these multicomponent forms being of interest. The published literature provides a limited number of new cocrystals and solvates, which is insufficient to obtain reliable structure–property relations (Cadden *et al.*, 2019 ▸). Ten cocrystals of AMG 517 were reported, accompanied with an evaluation of the physicochemical properties in order to correlate properties of various cocrystals (Stanton & Bak, 2008 ▸). Despite not obtaining structural data for most of the cocrystals, an interesting correlation of increasing cocrystal melting points with increasing guest molecule melting points was observed (Stepanovs *et al.*, 2015 ▸; Stanton & Bak, 2008 ▸; Aakeröy *et al.*, 2006 ▸). A different, more detailed analysis of cocrystal melting points across a broader system of compounds was performed by Schultheiss & Newman (2009 ▸), showing that 51% of the examined cocrystals had a melting point between those of the API and the guest molecule, 39% had a melting point lower than both the API and the guest molecule, 6% were higher than both, and 4% were same as the API or the guest molecule.

Most of the scientific literature about cocrystals and solvates currently available does not focus on structure–property relations or does not contain sufficiently large datasets to draw conclusions. Furthermore, introducing different guest molecules into a multicomponent crystal can completely change the crystal lattice. Even the properties of polymorphs, containing only a single compound with a different crystal arrangement, change significantly (Zvoníček *et al.*, 2018 ▸; Aguiar *et al.*, 1967 ▸; Aguiar & Zelmer, 1969 ▸; Bauer *et al.*, 2001 ▸). Therefore, by comparing multicomponent solid forms, changes in the crystal lattice are shown to impact the physicochemical properties to the same extent as the chemical nature of the guest molecule (de Moraes *et al.*, 2017 ▸).

## 2. Experimental

### 2.1. Intrinsic dissolution rate

The intrinsic dissolution rate (IDR) was determined using a Sirius inForm (Pion Inc. USA) device. IDR discs with a 6 mm diameter were prepared by compression of 40–60 mg of the tested material. The material was compressed at a constant load of 100 kg, relaxed for 1 min and compressed again at a constant load of 100 kg for a further 1 min. IDR measurements were performed in 40 ml phosphate buffer solution at pH 6.8 with the addition of 0.2% of sodium do­decyl sulfate (SDS) at a stirring speed of 100 rpm. UV spectra were recorded every 8 s using a 20 mm optical path length. Absorbance between wavelengths of 300 and 400 nm was used to evaluate the amount of API released at each time point. The IDR was calculated using a linear fit (*R* ^2^ > 0.90) of the experimental data over the minimum time frame of 2 min. Three measurement averages were used for the final IDR evaluation.

### 2.2. Differential scanning calorimetry

Samples for differential scanning calorimetry (DSC) measurements were weighed in an aluminium pan (∼10 mg). The pan was covered, and the measurement was carried out under a nitro­gen gas flow of 50 ml min^−1^. All measurements were performed on the TA Instruments Discovery DSC. The investigated temperatures ranged from 0 to 300°C at a heating rate of 10°C min^−1^ (amplitude = 0.8°C; period = 60 s).

### 2.3. Equilibrium solubility

Multicomponent form samples were analyzed using the Waters Acquity UPLC system equipped with a PDA detector (measured at 230 nm wavelength) and an Acquity BEH Phenyl column (100 × 2.1 mm; 1.7 µm). The temperature of the column was 45°C. The following gradient of 0.1% (*v*/*v*) phospho­ric acid/aceto­nitrile at a flow rate of 0.35 ml min^−1^ was used: steady state (0–0.5 min) followed by linear gradient from 70/30 to 20/80 (0.5–1.5 min) followed by steady state (1.5–2.5 min) and a linear gradient back to the starting conditions (2.5–3 min) followed by steady state for 2 min of re-equilibration. The injection volume was 1 µl. The data were processed using the *EMPOWER* software.

### 2.4. X-ray powder diffraction

The diffraction patterns were collected with the powder diffractometer device X’PERT PRO MPD PANalytical, a Cu *K*α X-ray beam (λ = 1.542 Å), 2–40° 2θ measured range, 45 kV excitation voltage, 40 mA anodic current, 0.01° 2θ step size and a 0.05 s step duration. The measurement was performed on a flat sample with an area:thickness ratio equal to 10:0.5 mm. The 0.02 rad Soller slits, 10 mm mask and 1/4° fixed anti-scattering slits were used to correct the primary beam. The irradiated area of the sample was 10 mm; programmable divergent slits were used. The 0.02 rad Soller slits and 5.0 mm anti-scattering slits were used to correct the secondary beam. The *HighScore Plus* software (Degen *et al.*, 2014 ▸) was used to process the diffraction patterns.

### 2.5. Raman spectroscopy

Samples for Raman spectroscopy were measured in HPLC glass vials in an FT-Raman RFS100/S spectrometer device with a germanium detector (Bruker Optics, Germany). The wavelength of the Nd:YAG laser was 1064 nm. The range measured was 4000 to 200 cm^−1^, with a spectral resolution of 4.0 cm^−1^. Data were obtained either at 64 or 128 accumulations of the measured spectra. The software *OMNIC* and *OPUS* were used to process the Raman spectra.

### 2.6. Single-crystal X-ray diffraction

X-ray analyses of the apremilast multicomponent forms were performed either at 95 K using a SuperNova diffractometer with a micro-focus sealed tube, mirror-collimated Cu *K*α radiation (λ = 1.54184 Å) and CCD detector Atlas S2; or at 120 K on an Xcalibur, Gemini ultra diffractometer using Cu *K*α radiation (λ = 1.54178 Å) from a fine-focus sealed X-ray tube with a graphite monochromator and CCD detector Atlas S2.

The data reduction and absorption correction were carried out with *CrysAlisPro* (Rigaku Oxford Diffraction, 2019 ▸). The structure was solved by charge flipping methods using the *Superflip* software (Palatinus & Chapuis, 2007 ▸) and refined by full matrix least squares on the *F*-squared value using the *Crystals* software (Betteridge *et al.*, 2003 ▸). The *MCE* software (Rohlíček & Hušák, 2007 ▸) was used for visualization of residual electron density maps. According to common practice, hydrogen atoms attached to carbon atoms were assigned geometrically with *U*_iso_ (H) in the range 1.2–1.5 *U*_eq_ of the parent atom (C).

## 3. Results and discussion

**Figure 1:**
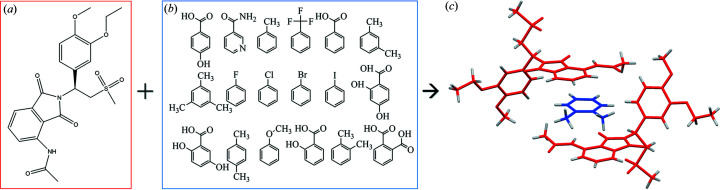
(*a*) Molecule of apremilast, (*b*) guest molecules, (*c*) crystal structure of cocrystals and solvates (*o*-xylene solvate is shown as an example).

This study reports 15 new cocrystals and solvates of the pharmaceutical molecule apremilast, which is used for the treatment of psoriasis and psoriatic arthritis (Zerilli & Ocheretyaner, 2015 ▸; Afra *et al.*, 2019 ▸). The structures of all new forms were determined by single-crystal X-ray diffraction (SCXRD) and their physicochemical properties were evaluated. The properties of five known, similar multicomponent apremilast forms (Wu *et al.*, 2017 ▸; Jirát *et al.*, 2019 ▸, 2020 ▸) were included to provide a slightly larger dataset to draw more precise conclusions. Please note, the pharmaceutical relevance of the guest molecule was not considered in order to enlarge the dataset. New cocrystals of apremilast crystallized with 2,4-di­hydroxy­benzoic acid (24HB_C_), 2,5-di­hydroxy­benzoic acid (25HB_C_), 4-hy­droxy­benzoic acid (4HB_C_), nicotinamide (NCA_C_) and salicylic acid (SCA_C_); and solvates with anisole (ANI_S_), bromo­benzene (BRB_S_), chloro­benzene (CLB_S_), hexa­fluoro­benzene (HFBs), iodo­benzene (IOB_S_), mesitylene (MST_S_), *m*-xylene (MXY_S_), *p*-xylene (PXY_S_) and α,α,α-tri­fluoro­toluene (TFT_S_). Other multicomponent forms from the literature (Wu *et al.*, 2017 ▸; Jirát *et al.*, 2019 ▸, 2020 ▸) and used in this study crystallized with phthalic acid (PHT_C_), benzoic acid (BAC_C_), toluene (TOL_S_), *o*-xylene (OXY_S_) and fluoro­benzene (FLB_S_). Their respective chemical structures together with that of apremilast are presented in Fig. 1 ▸.

**Figure 2:**
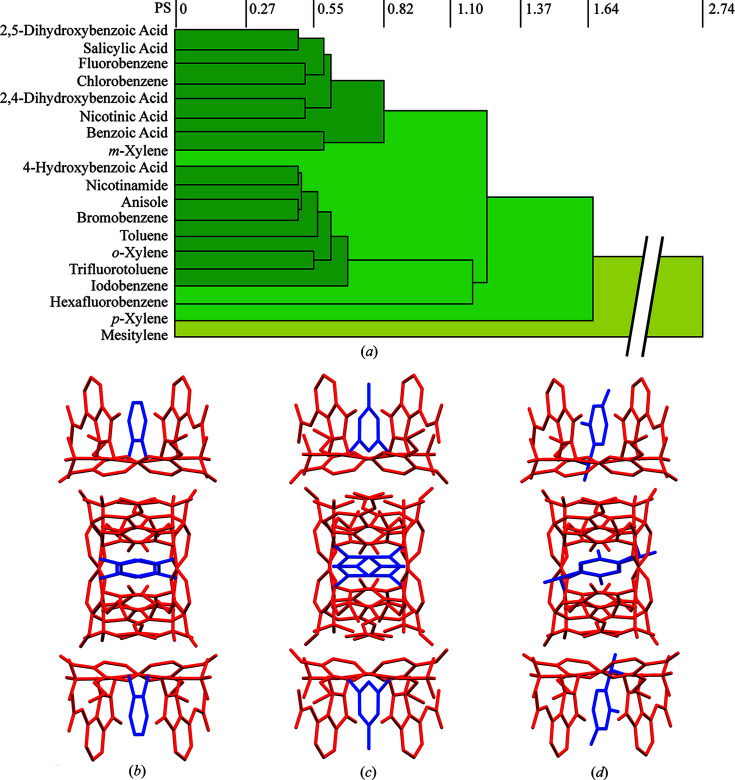
(*a*) Dendrogram showing the packing similarity between all forms studied here, produced using *CrystalCMP*. (*b*) 2,4-Di­hydroxy­benzoic acid cocrystal, (*c*) bromo­benzene solvate and (*d*) mesitylene solvate, all showing similar crystal packing displayed along the *c* axis. Hydrogen atoms have been omitted for clarity.

Since the structural analysis of similar apremilast solid forms is already well discussed in the scientific literature (Jirát *et al.*, 2020 ▸; Dudek *et al.*, 2019 ▸), and our new crystal structures are isostructural, only key aspects of these systems are mentioned below (crystallographic details are provided in the supporting information). All new solid forms crystallized in the tetragonal system with the *P*4_1_2_1_2 space group and cell parameters (*a* ≃ 13 Å, *c* ≃ 29 Å). The ratio between the API and the guest molecule is 2:1. The observed pattern, which is similar for all investigated forms, is the π–π stacking interaction of the guest molecule benzene ring with the phthalimide group of the apremilast molecule. It is important to emphasize that all 19 cocrystals and solvates used throughout this study are isostructural (see Fig. 2 ▸).

All prepared forms are compared in Fig. 2 ▸(*a*) using the *CrystalCMP* software (Rohlíček *et al.*, 2016 ▸) and the results show high packing similarity between all 19 forms used (a detailed description of *CrystalCMP* is given in the supporting information). The two forms with the biggest differences in crystal packing are shown in Figs. 2 ▸(*b*) and 2(*d*) and one in between [Fig. 2 ▸(*c*)] for reference. It is immediately evident that, despite choosing the most different forms, they still exhibit very similar crystal packing. Such a level of crystal packing similarity is very rare for multicomponent forms of APIs. It is interesting from a crystallography point of view, and it presents a close-to-ideal system to study structure–property relations.

The isostructurality of these samples ensures that the impact of the crystal lattice will be minimal or negligible. This allows for a more direct correlation between the properties of the guest molecule and the properties of the respective multicomponent form within a large and isostructural dataset. The physicochemical properties evaluated were thermal stability (melting temperature, *T*_m_), IDR and equilibrium solubility (EqSol), which are commonly determined for new solid forms and are crucial for pharmaceutical development.

**Figure 3:**
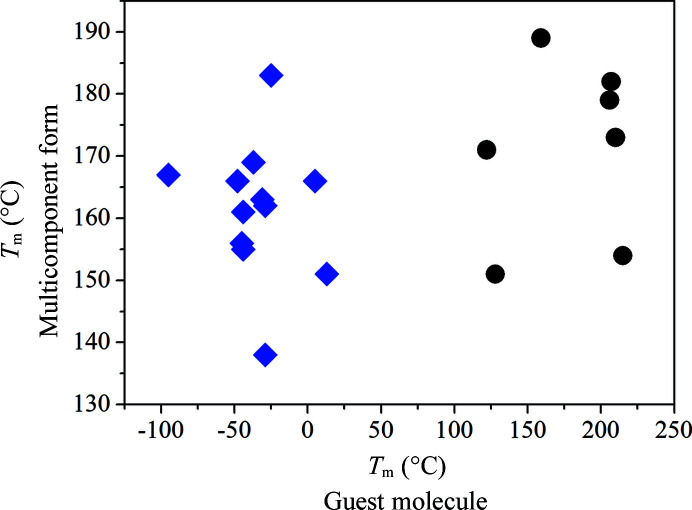
Melting points of multicomponent forms and guest molecules. Cocrystals are marked with black circles and solvates with blue diamonds. A linear fit of the data gives an *R* ^2^ of 0.1366.

First, we evaluated the melting temperatures of the multicomponent solids, which range from 139°C (tri­fluoro­toluene solvate) to 190°C (salicylic acid cocrystal); for more details, refer to the supporting information. DSC curves obtained confirmed phase purity of the prepared multicomponent forms and were further used to examine the correlation between melting points of the multicomponent solids and their guest molecules (Fig. 3 ▸).

Data presented in Fig. 3 ▸ indicate there is no relation between the melting points of the guest molecules and the multicomponent forms. The melting points of the solvates and cocrystals were further compared in their respective groups (solvates with solvents and cocrystals with coformers), but still no relation was observed. The melting points were further studied revealing that 1 in 19 (∼5%) multicomponent forms has a lower melting point than both apremilast and the guest molecule, 7 in 19 (∼37%) have a melting point between those of apremilast and the guest molecule, and 11 in 19 (∼59%) have a higher melting point than apremilast and the guest molecule. These findings depart from the majority of the experimental data published in the literature (Schultheiss & Newman, 2009 ▸; Stepanovs *et al.*, 2015 ▸; Stanton & Bak, 2008 ▸; Aakeröy *et al.*, 2006 ▸). However, there are studies that report similar results to ours (Stanton *et al.*, 2009 ▸). This suggests that there will not be a simple and universal correlation between melting points of multicomponent forms and their guest molecules. It is possible to discover correlations in smaller systems, while considering the chemical nature of the guest molecules. However, for larger datasets and various compounds this might not be the case. In fact, as concluded from Fig. 3 ▸, it was not possible to find any relation despite the isostructurality of the prepared apremilast multicomponent forms. The interactions of guest molecules with apremilast molecules are different for each guest molecule despite occupying the same position in the crystal lattice; this is due to their changing chemical nature. The different intermolecular interactions can cause differences in thermal behaviour and make the idea of simple correlations invalid across broad systems. On the other hand, a deeper understanding of the intermolecular interactions, with improving crystal lattice calculations and better testing datasets in recent years might lead to successful predictions and correlations of melting points in the future.

The IDR was measured and evaluated for 17 samples (the IDR measurement was unsuccessful for iodo­benzene solvent and the salicylic acid cocrystal). The IDR ranges from 5 to 159 µg min^−1^ cm^−2^, the lowest IDR was observed for *o*-xylene solvate and the highest for 2,5-di­hydroxy­benzoic acid cocrystal. The up to tenfold increase in IDR for some of the new forms is very significant compared with the solid-state form used in the original drug product, which has an IDR of ∼14 µg min^−1^ cm^−2^ (for more details refer to the supporting information). The IDR values were compared with the melting temperatures of the samples.

**Figure 4:**
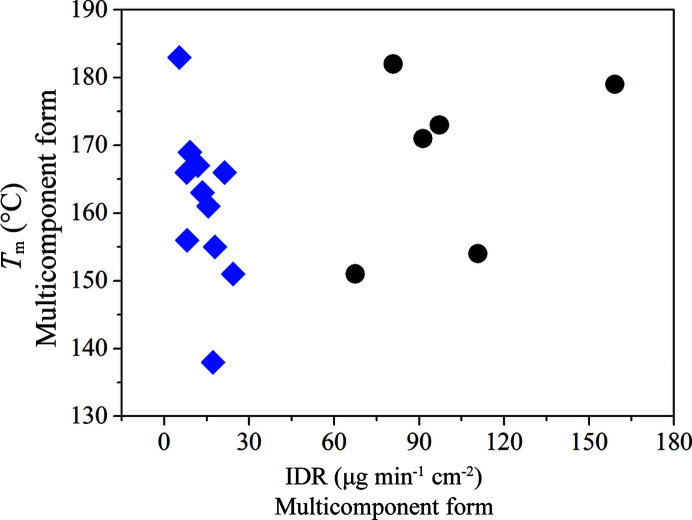
Correlation of melting temperatures and IDRs of the multicomponent forms. Cocrystals are marked with black circles and solvates with blue diamonds. A linear fit of the data gives an *R* ^2^ of 0.0881.

Data presented in Fig. 4 ▸ indicate that there is no relation between IDR and the melting points of these isostructural apremilast forms. Stanton and Bak examined similar correlations of IDR and melting temperature for cocrystals and discovered only a very weak correlation (Stanton & Bak, 2008 ▸) or no correlation at all (Stanton *et al.*, 2009 ▸), which is mostly in agreement with the data presented here. These outcomes suggest that it is not possible to estimate the dissolution rate of multicomponent forms based on their melting temperature. The IDRs of the solvates are significantly lower compared with those of the cocrystals. This difference might be partially explained by the differences in polarity of the guest molecules. The polarity of the guest molecules presented ranges widely from non-polar or slightly polar, such as mesitylene or xylenes, to very polar molecules, such as benzoic or salicylic acid. More polar molecules might display higher IDRs due to their ability to compete with hydrogen bonds of water molecules, thus being more readily soluble. This phenomenon affects the IDR of a multicomponent form as the guest molecule is the integral part.

It is important to note that the solid phases of the measured samples were evaluated before and after the IDR measurement and a solid-state transformation did not occur in any of these cases. This shows high phase stability of the apremilast multicomponent forms in the dissolution media for the time period of the measurement (∼1 h).

Since the IDR and melting points are not correlated, it is convenient to examine whether the IDR relates to the EqSol of the guest molecule in the same dissolution medium or the EqSol of the new multicomponent forms.

In this case, it is possible to observe a correlation between the IDR of the multicomponent form with the EqSol of both the multicomponent form and the guest molecule. The correlation between the IDR of the multicomponent form and EqSol of its guest molecule might be of strong interest. This would allow us to estimate the IDR of the multicomponent form in advance which would be beneficial in the design. Such an estimation of the solid-state form IDR prior to its formation could save in costs and experimental efforts during drug development. Especially as IDR is one of the crucial parameters that impacts bioavailability.

**Figure 5:**
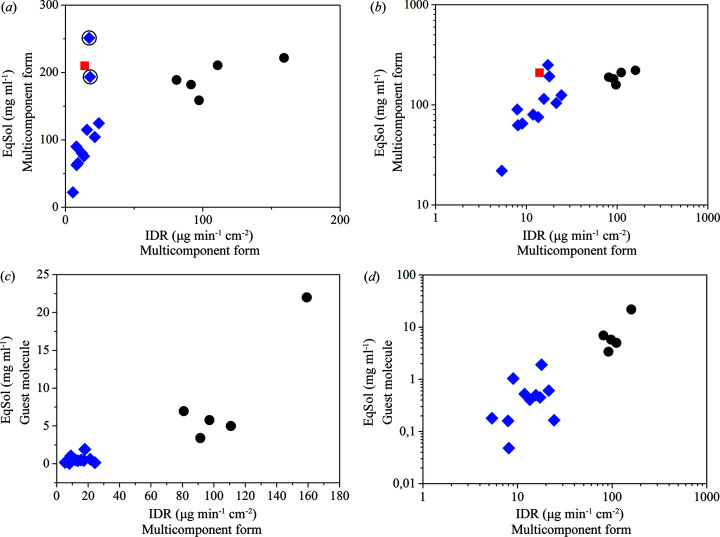
(*a*) and (*b*) Correlation of IDR and EqSol of multicomponent forms. (*c*) and (*d*) Correlation of IDR of the multicomponent forms and EqSol of the respective guest molecules. Note, nicotinamide is not included here since its EqSol is several orders of magnitude higher compared with the EqSol values of other guest molecules. Cocrystals are marked with black circles, solvates with blue diamonds and pure apremilast with red squares. (*a*) and (*c*) Linear plots; (*b*) and (*d*) log–log plots. A logarithmic fit of the data in (*a*) gives an *R* ^2^ of 0.56 with outliers and an *R* ^2^ of 0.94 without outliers marked with black circles. A linear fit of the data in (*b*) gives an *R* ^2^ of 0.76.

Since this is a real system, deviations from the correlations are observed for some samples despite the high similarity of the prepared multicomponent forms. Fluoro­benzene and tri­fluoro­toluene solvates deviate from the correlation and are marked with black circles in Fig. 5 ▸(*a*). This might be caused by the high electronegativity of the fluorine atoms, which can impact intermolecular interactions within the crystal lattice of individual multicomponent forms. These interactions play a major role and might cause the deviations from the observed relations. It is necessary to study these interactions further as they may account for the behaviour of the samples that do not fit the trend.

## 4. Conclusions

We report 15 new, isostructural multicomponent solids of apremilast, both cocrystals and solvates, with solved crystal structures from SCXRD. With an additional 5 similar and already published forms, we created a large and unique dataset of 19 isostructural solid forms. This dataset provides the possibility to systematically explore structure–property relations within multicomponent isostructural solids. The physicochemical properties evaluated were melting point, intrinsic dissolution rate and thermodynamic solubility, which are commonly characterized for new solids and are important for pharmaceutical drug development. We observed no relation between the melting points of the multicomponent forms and their guest molecules. In addition, no correlation was found between the melting points and the intrinsic dissolution rate of the multicomponent forms. However, a considerable correlation was found between the intrinsic dissolution rate of the multicomponent forms and their solubility as well as the solubility of their guest molecules. The correlation with the solubility of guest molecules is particularly interesting as it could help with the design of multicomponent forms with desirable properties. Overall, it is clear that discovering universal and simple correlations across a broad system of compounds and structures is a difficult task if not an impossible one. Even in this almost ideal and so far unique system, there are a few deviations from the observed correlations. The contribution of the calculation approach will shed new light on the specific differences in the intermolecular interactions, and obtaining more well defined datasets would be beneficial for future progress in the crystal engineering field. For now, as far as we know, this is the most reliable experimental dataset available and correlations drawn for non-ionized multicomponent solid forms.

## 5. Related literature

The following references are cited in the supporting information: Dudek *et al.* (2018 ▸); Wang *et al.* (2018 ▸); Chiou *et al.* (1977 ▸); Valvanix *et al.* (1981 ▸); Isao *et al.* (1982 ▸); Freire *et al.* (2005 ▸); Van Arnum (2000 ▸); Nian (2016 ▸); Yalkowsky & Dannenfleser (1992 ▸); Yalkowsky & He (1992 ▸); Yalkowsky *et al.* (2010 ▸). 

## Supplementary Material

Crystal structure: contains datablock(s) Apremilast24dihydroxybenzoicacid, apremilast25dihydrbenzoic, Apremilast_4_hydroxybenzoic_acid, apremilastanisol, apremilast_brombenzen, Apremilast_chlorbenzen, global, ApreHexa, apremilastiodobenzene, apremilastmesitilene, apremilastmetaxylene, apremilastnicotinamide, apremilastniacin, Apremilast_paraxylene, apremilastsalycilic, Apremilasttrifluorotoluene. DOI: 10.1107/S2052252522005577/ed5027sup1.cif

Structure factors: contains datablock(s) ApreHexa. DOI: 10.1107/S2052252522005577/ed5027sup2.hkl

Structure factors: contains datablock(s) Apremilast24dihydroxybenzoicacid. DOI: 10.1107/S2052252522005577/ed5027sup3.hkl

Structure factors: contains datablock(s) Apremilast_4_hydroxybenzoic_acid. DOI: 10.1107/S2052252522005577/ed5027sup4.hkl

Structure factors: contains datablock(s) Apremilast_chlorbenzen. DOI: 10.1107/S2052252522005577/ed5027sup5.hkl

Structure factors: contains datablock(s) Apremilast_paraxylene. DOI: 10.1107/S2052252522005577/ed5027sup6.hkl

Structure factors: contains datablock(s) Apremilasttrifluorotoluene. DOI: 10.1107/S2052252522005577/ed5027sup7.hkl

Structure factors: contains datablock(s) apremilast25dihydrbenzoic. DOI: 10.1107/S2052252522005577/ed5027sup8.hkl

Structure factors: contains datablock(s) apremilast_brombenzen. DOI: 10.1107/S2052252522005577/ed5027sup9.hkl

Structure factors: contains datablock(s) apremilastanisol. DOI: 10.1107/S2052252522005577/ed5027sup10.hkl

Structure factors: contains datablock(s) apremilastiodobenzene. DOI: 10.1107/S2052252522005577/ed5027sup11.hkl

Structure factors: contains datablock(s) apremilastmesitilene. DOI: 10.1107/S2052252522005577/ed5027sup12.hkl

Structure factors: contains datablock(s) apremilastmetaxylene. DOI: 10.1107/S2052252522005577/ed5027sup13.hkl

Structure factors: contains datablock(s) apremilastniacin. DOI: 10.1107/S2052252522005577/ed5027sup14.hkl

Structure factors: contains datablock(s) apremilastnicotinamide. DOI: 10.1107/S2052252522005577/ed5027sup15.hkl

Structure factors: contains datablock(s) apremilastsalycilic. DOI: 10.1107/S2052252522005577/ed5027sup16.hkl

Supporting tables, figures and experimental details. DOI: 10.1107/S2052252522005577/ed5027sup17.pdf

CCDC references: 2049269, 2049270, 2049271, 2049272, 2049273, 2049274, 2049275, 2049276, 2049277, 2049278, 2049279, 2049280, 2049281, 2049282, 2071132
