# Supplementary material for: Structure–property relations of a unique and systematic dataset of 19 isostructural multicomponent apremilast forms
Source: IUCrJ. 2022 Jun 15;9(Pt 4):508–15. doi: 10.1107/S2052252522005577 (PMC9252161; doi:10.1107/S2052252522005577)
Supplement: Supplementary file 17 [file m-09-00508-sup17.pdf]

IUCrJ

Volume 9 (2022)

Supporting information for article:

Structure–property relations of a unique and systematic dataset of 19 isostructural multicomponent apremilast forms

Jan Jirát, Martin Babor, Luděk Ridvan, Eliška Skořepová, Michal Dušek and Miroslav Šoóš

Structure-Property Relation of Unique and Systematic Dataset of 19 Isostructural Multicomponent Apremilast Forms

Jan Jirát[a,c], Martin Babor[b,c], Luděk Ridvan[c], Eliška Skořepová[a,d], Michal Dušek[d], Miroslav Šoóš[a]

[a] *Department of Chemical Engineering, University of Chemistry and Technology, Technická 3, 166 28 Prague 6 – Dejvice, Czech Republic*

[b] *Department of Solid State Chemistry, University of Chemistry and Technology, Technická 5, 166 28 Prague 6 – Dejvice, Czech Republic*

[c] *Zentiva, k.s., U kabelovny 130, 10237, Prague 10, Czech Republic*

[d] *Institute of Physics of the Czech Academy of Sciences, Na Slovance 2, 182 00 Prague 8, Czech Republic*

Crystallography

All presented apremilast multicomponent forms crystallized in tetragonal system with *P* 4_1 2_1 2 space group. This system is unique due to all prepared forms being isostructural. More crystallography details and details from structure solution is in Tab. S1.

Tab. S1: Crystallography details and structure solution details.

Number	1	2	3	4
Guest molecule	2,4-Dihydroxybenzoic acid	2,5-Dihydroxybenzoic acid	4-Hydroxybenzoic acid	Nicotinamide
CCDC deposit number	2049271	2049273	2049272	2049278
Empirical formula	2(C22 H24 N2 O7 S), C7 H4	2(C22 H24 N2 O7 S), C7 H5	2(C22 H24 N2 O7 S), C7 H3	2(C22 H24 N2 O7 S), C6 H4
Diffractometer	SuperNova	SuperNova	SuperNova	SuperNova
Wavelength (Å)	1.54180	1.54180	1.54180	1.54180
mol. weight (g/mol)	1073.09	1074.09	1056.08	1041.10
Crystal system	Tetragonal	Tetragonal	Tetragonal	Tetragonal
Space group	P 41 21 2	P 41 21 2	P 41 21 2	P 41 21 2
a (Å)	12.9189(1)	12.9170(1)	12.9076(1)	12.8876(1)
b (Å)	12.9189(1)	12.9170(1)	12.9076(1)	12.8876(1)
c (Å)	29.4056(1)	29.3859(2)	29.4670(2)	29.4547(2)
α (°)	90	90	90	90
β (°)	90	90	90	90
γ (°)	90	90	90	90
Volume ($Å^3$)	4907.74(8)	4903.01(8)	4909.38(8)	4892.14(8)
Z	4	4	4	4
Dens.(calc.) (g/cm^3)	1.452	1.455	1.429	1.413
Abs. coeff. (mm^{-1})	1.689	1.691	1.664	1.641
F(000)	2248.0	2252.0	2212.0	2184.0
Crystal size (mm^3)	0.152x0.173x0.366	0.176x0.250x0.441	0.116x0.136x 0.266	0.158x0.238x0.316
Crystal description	prism	block	block	block
θ range (°)	3.737;67.935	3.738;67.744	3.739;67.760	3.744;67.663
Refl. collected	94480	88004	84206	90858
Indep. reflections	4464 [Rint =0.033]	4443 [Rint=0.052]	4433 [Rint=0.042]	4429 [Rint=0.039]
Reflections obs.	4409	4287	4320	4359
Criterion for obs.	[I > 2.0σ(I)]	[I > 2.0σ(I)]	[I > 2.0σ(I)]	[I > 2.0σ(I)]
Completeness to θ (°)	0.999 to 67.935	1.000 to 67.744	0.996 to 67.760	1.000 to 67.663
Absorption corr.	multi-scan	multi-scan	multi-scan	multi-scan
Min. and max. transm.	0.739 and 0.774	0.632 and 0.743	0.762 and 0.824	0.657 and 0.772
Data / restraints / param.	4462/62/363	4443/12/354	4433/62/356	4429/57/350
Goodness-of-fit on F^2	0.9986	1.0044	1.0266	1.0243
Fin. R indices [I>2sigma(I)]	R1=0.0479, wR2=0.1354	R1=0.0389, wR2=0.1011	R1=0.0504, wR2=0.1444	R1=0.0389, wR2=0.1056

R indices (all data)	R1=0.0482, wR2=0.1358	R1=0.0401, wR2=0.1022	R1=0.0513, wR2=0.1456	R1=0.0393, wR2=0.1060
Fin. diff. ρmax (e^- /$Å^{-3}$)	0.64 and -0.61	0.62 and -0.35	0.70 and -0.86	0.54 and -0.47
Flack parameter	0.02(3)	0.01(2)	-0.000(2)	0.01(2)
Temperature of measurement (K)	120	120	120	120

5	6	7	8	9
Nicotinic Acid	Salicylic acid	Anisole	Chlorobenzene	Bromobenzene
2049274	2049282	2049270	2049269	2049275
2(C22 H24 N2 O7 S), C6 H5 N O2	2(C22 H24 N2 O7 S), C7 H5	2(C22 H24 N2 O7 S), C7 H8	4(C22 H24 N2 O7 S), C12 H9 Cl2	2(C22 H24 N2 O7 S), C6 H5 Br
SuperNova	SuperNova	SuperNova	SuperNova	SuperNova
1.54184	1.54180	1.54180	1.54180	1.54180
1044.09	1058.09	1029.12	2066.06	1077.98
Tetragonal	Tetragonal	Tetragonal	Tetragonal	Tetragonal
P 41 21 2	P 41 21 2	P 41 21 2	P 41 21 2	P 41 21 2
12.9268(1)	12.9133(1)	12.8885(1)	12.8788(1)	12.8625(1)
12.9268(1)	12.9133(1)	12.8885(1)	12.8788(1)	12.8625(1)
29.3204(2)	29.3418(1)	29.5227(1)	29.3261(1)	29.5499(1)
90	90	90	90	90
90	90	90	90	90
90	90	90	90	90
4899.50(8)	4892.84(8)	4904.12(8)	4864.13(8)	4888.85(8)
4	4	4	2	4
1.416	1.436	1.394	1.411	1.465
1.649	1.669	1.616	2.110	2.554
2192.0	2220.0	2168.0	2166.0	2240.0
0.210x0.284x0.294	0.363x0.387x0.676	0.138x0.211x0.221	0.170x0.224x0.360	0.090x0.167x0.218
block	block	block	block	block
3.737;67.452	3.740;67.621	3.742;67.603	4.570;67.616	3.748;67.548
83935	93647	61003	45672	92129
4406 [Rint=0.060]	4419 [Rint=0.027]	4440 [Rint=0.031]	4375 [Rint=0.035]	4425 [Rint=0.033]
4270	4398	4342	4293	4321
[I > 2.0σ(I)]	[I > 2.0σ(I)]	[I > 2.0σ(I)]	[I > 2.0σ(I)]	[I > 2.0σ(I)]
0.999 to 67.452	0.998 to 67.621	1.000 to 67.603	0.996 to 66.264	1.000 to 67.548
multi-scan	multi-scan	multi-scan	multi-scan	multi-scan
0.647 and 0.707	0.484 and 0.546	0.735 and 0.800	0.595 and 0.699	0.629 and 0.795
4406/20/353	4419/1/354	4440/8/333	4375/120/353	4425/61/325
0.9999	1.0911	1.1032	0.9885	0.9938
R1=0.0362, wR2=0.0922	R1=0.0301, wR2=0.0754	R1=0.0323, wR2=0.0453	R1=0.0324, wR2=0.0889	R1=0.0735, wR2=0.2128
R1=0.0374, wR2=0.0932	R1=0.0302, wR2=0.0755	R1=0.0332, wR2=0.0459	R1=0.0330, wR2=0.0895	R1=0.0747, wR2=0.2147
0.29 and -0.31	0.44 and -0.40	0.76 and -0.35	0.74 and -0.48	2.25 and -2.35

-0.003(19)	0.028(15)	0.010(16)	-0.002(15)	0.012(3)
120	120	120	120	120

10	11	12	13	14
Iodobenzene	m-Xylene	p-Xylene	Mesitylene	Trifluorotoluene
2049276	2049277	2049280	2049279	2049281
2(C22 H24 N2 O7 S), C6 H5 I	C22 H24 N2 O7 S, 0.5(C8 H10)	2(C22 H24 N2 O7 S), C8 H13	2(C22 H24 N2 O7 S), C9 H12	2(C22 H24 N2 O7 S), C7 H6 F3
Gemini	SuperNova	SuperNova	SuperNova	SuperNova
1.54180	1.54180	1.54180	1.54180	1.54180
1124.98	513.57	1030.17	1041.17	1068.10
Tetragonal	Tetragonal	Tetragonal	Tetragonal	Tetragonal
P 41 21 2	P 41 21 2	P 41 21 2	P 41 21 2	P 41 21 2
12.9104(1)	12.9696(1)	13.1850(1)	13.2792(1)	12.9492(1)
12.9104(1)	12.9696(1)	13.1850(1)	13.2792(1)	12.9492(1)
29.4495(1)	29.2499(1)	28.5930(1)	28.2737(1)	29.6806(3)
90	90	90	90	90
90	90	90	90	90
90	90	90	90	90
4908.60(8)	4920.14(8)	4970.73(8)	4985.70(8)	4976.90(9)
4	8	4	4	4
1.522	1.387	1.377	1.387	1.426
6.529	1.594	1.577	1.580	1.688
2312.0	2168.0	2180.0	2200.0	2236.0
0.121x0.136x0.190	0.245x0.276x0.330	0.110x0.229x0.279	0.309x0.348x0.487	0.178x0.189x0.324
block	block	plate	block	block
3.738;76.331	3.728;67.595	3.692;67.660	3.677;67.664	3.724;67.641
86093	100964	97817	90441	119531
5141 [Rint=0.030]	4454 [Rint=0.037]	4498 [Rint=0.031]	4516 [Rint=0.035]	4502 [Rint=0.047]
5108	4439	4440	4479	4478
[I > 2.0σ(I)]	[I > 2.0σ(I)]	[I > 2.0σ(I)]	[I > 2.0σ(I)]	[I > 2.0σ(I)]
0.999 to 76.331	0.999 to 67.595	0.999 to 67.660	1.000 to 67.664	0.999 to 67.641
multi-scan	multi-scan	multi-scan	multi-scan	multi-scan
0.396 and 0.454	0.620 and 0.677	0.681 and 0.841	0.543 and 0.614	0.716 and 0.740
5141/16/329	4453/125/362	4498/46/333	4516/6/337	4502/185/381
0.9968	1.1151	0.9300	0.8635	0.9740
R1=0.0356, wR2=0.0792	R1=0.0356, wR2=0.0792	R1=0.0274, wR2=0.0595	R1=0.0223, wR2=0.0601	R1=0.0670, wR2=0.1605
R1=0.0358, wR2=0.0794	R1=0.0358, wR2=0.0794	R1=0.0277, wR2=0.0598	R1=0.0224, wR2=0.0603	R1=0.0672, wR2=0.1606
0.99 and -1.74	0.20 and -0.23	0.32 and -0.78	0.21 and -0.26	0.38 and -0.57
-0.004(4)	0.03(2)	-0.0000(7)	-0.003(11)	0.06(4)
95	120	120	120	120

15
Hexafluorobenzene
2071132
2(C22 H24 N2 O7 S), (C6 F6)
SuperNova
1.54184
1107.10
tetragonal
P 41 21 2
12.84250(10)
12.84250(10)
29.4534(2)
90
90
90
4857.74(6)
4
1.514
1.844
2296.0
0.17x0.252x0.34
block
3.75; 74.27
41296
4928 [Rint = 0.0182]
4897
[I > 2.0σ(I)]
1.000 to 74.27
analytical
0.601 and 0.731
4928/93/349
1.66
R1=0.0226, wR2=0.0716
R1=0.0228, wR2=0.1358
0.16 and -0.21
0.004(9)
120

Forms in Tab. S1 are new and published for the first time in this journal. However, to enlarge dataset for correlations, five similar forms of apremilast were added to the dataset. In particular, toluene

solvate (CCDC: 1546012), o-xylene solvate (CCDC: 1985110) and fluorobenzene (CCDC: 1985111) solvate and phthalic acid cocrystal (CCDC: 1991669) and benzoic acid cocrystal (CCDC: 1911987). More information about their crystal structures can be found in Cambridge Structural Database under the mentioned CCDC numbers. Note that answers to B level alerts in checkcif are included in cif files.

Materials and Methods

Apremilast was kindly provided by Zentiva, k.s., as form B (which is used in the original drug product[1]). The solvents and crystallization partners were obtained from various commercial suppliers and were used as received, without any further purification.

Screening of multicomponent forms of apremilast (single crystal preparation)

Screening was based on available data in scientific literature[5-7]. It becomes immediately obvious that the rational way of screening is searching for small aromatic molecules (substituted benzene rings) in 2:1 molar ratio (apremilast:guest molecule). 21 molecules were chosen for the screening (Tab. S2). Different approaches were used for screening of cocrystals and screening of solvates. While screening for cocrystals, 50 mg of apremilast was mixed with an appropriate amount of the coformer (2:1 molar ratio), dissolved in acetone or methanol and let to freely evaporate. Sufficient single crystals were collected and measured. While screening for solvates, 50 mg of apremilast was dissolved in 200 µl of tetrahydrofuran (THF). After complete dissolution, 20 µl of chosen solvent was added into the solution. Since the boiling point of THF is lower compared to chosen solvents, THF evaporated first and apremilast crystallized into the chosen solvent. The rest of the solvent was let to freely evaporate, and sufficient amount of crystals was collected and measured.

Tab. S2: Molecules chosen for the multicomponent form screening. Molecules marked red resulted in physical mixture of both compounds, green resulted in cocrystal/solvate. Number in the bracket refers to a preparation method of scaled up samples (methods are described further in the text).

Chemical compound		
4-Hydroxybenzoic acid (1)	Chlorobenzene (2)	Nicotinamide (3)
2,4-Dihydroxybenzoic acid (1)	Fluorobenzene (2)	o-Xylene (2)
2,5-Dihydroxybenzoic acid (1)	Gallic acid (-)	Paracetamol (-)
Acetylsalicylic acid (-)	Iodobenzene (2)	p-Xylene (2)
Anisole (2)	Isonicotinamide (-)	Salicylic acid (1)
Benzoic acid (1)	Mesitylene (2)	Trifluorotoluene (2)
Bromobenzene (2)	m-Xylene (2)	Nicotinic acid (-)
Hexafluorobenzene (2)		

18 out of the 22 tested molecules resulted in the formation of multicomponent form. Size of the guest molecule appears to be crucial parameter[8].

Scale up of apremilast multicomponent forms

Scale up to several grams of the new forms was necessary for the measurement and evaluation of properties. Three different approaches were applied during the scale up.

1) 8 ml of methyl ethyl ketone (MEK) was saturated with chosen coformer at room temperature. The solution was filtered and then heated to 60 °C. 1.2 mg of apremilast was added at 60 °C and let to cool down. Crystallization occurred and the samples were mixed for 24 hours, filtered, and washed with methanol followed by drying (12 h, 40 °C, 100 mbar).
2) 1.2 mg of apremilast was dissolved in approx. 10 ml of THF. 2 ml of chosen solvent was added to the solution and resulting mixture was let to freely evaporate. The product was dried (2 h, 30 °C, 100 mbar) after the evaporation.
3) 2 ml of water was saturated with nicotinamide at room temperature. The solution was filtered and then heated to 60 °C. 1.2 mg of apremilast was added at 60 °C and let to cool down. Crystallization occurred and the samples was mixed for 24 hours, filtered, and washed with methanol, dried (12 h, 40 °C, 100 mbar).

All prepared samples were checked by standard solid-state analysis (XRPD, Raman, DSC, NMR) and their powder diffraction pattern was compared to pattern generated from corresponding crystal structures. The solid phase purity was confirmed for all samples.

Which method was used for preparation of each sample is stated in Tab. S2.

Note that for nicotinic acid the preparation of higher amount of the cocrystal was unsuccessful. Therefore, it was excluded from the correlations.

Results

Additional supporting data that was not shown in the manuscript is presented in this section in a form of figures and tables.

XRPD

XRPD patterns were measured and evaluated to determine formation of a new multicomponent form. Further, the XRPD patterns of the material used for the measurement and evaluation of properties was compared with patterns calculated from solved crystal structures. This ensures that structure could be properly linked with its properties (Fig. S1).

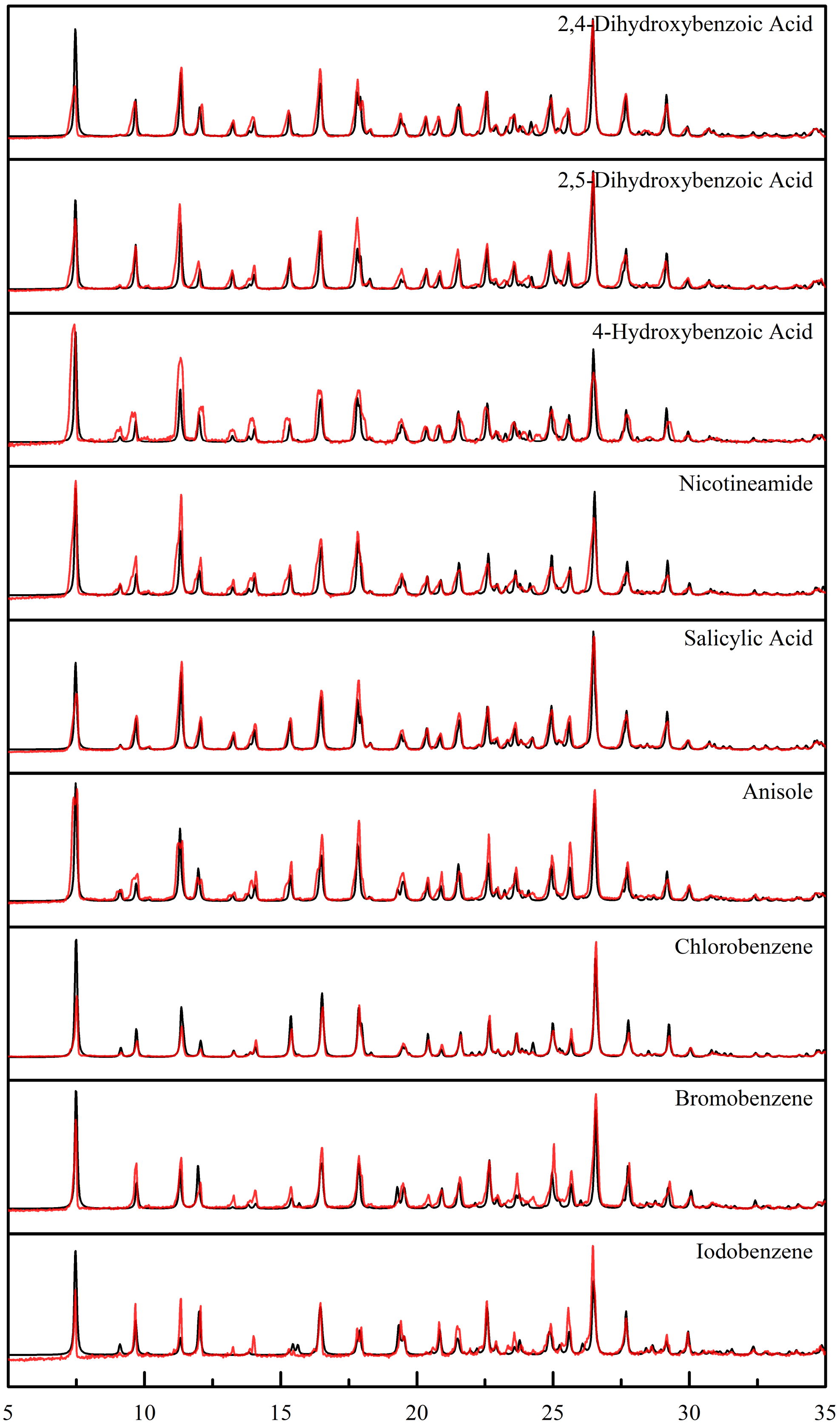
2,4-Dihydroxybenzoic Acid
2,5-Dihydroxybenzoic Acid
4-Hydroxybenzoic Acid
Nicotineamide
Salicylic Acid
Anisole
Chlorobenzene
Bromobenzene
Iodobenzene
5
10
15
20
25
30
35

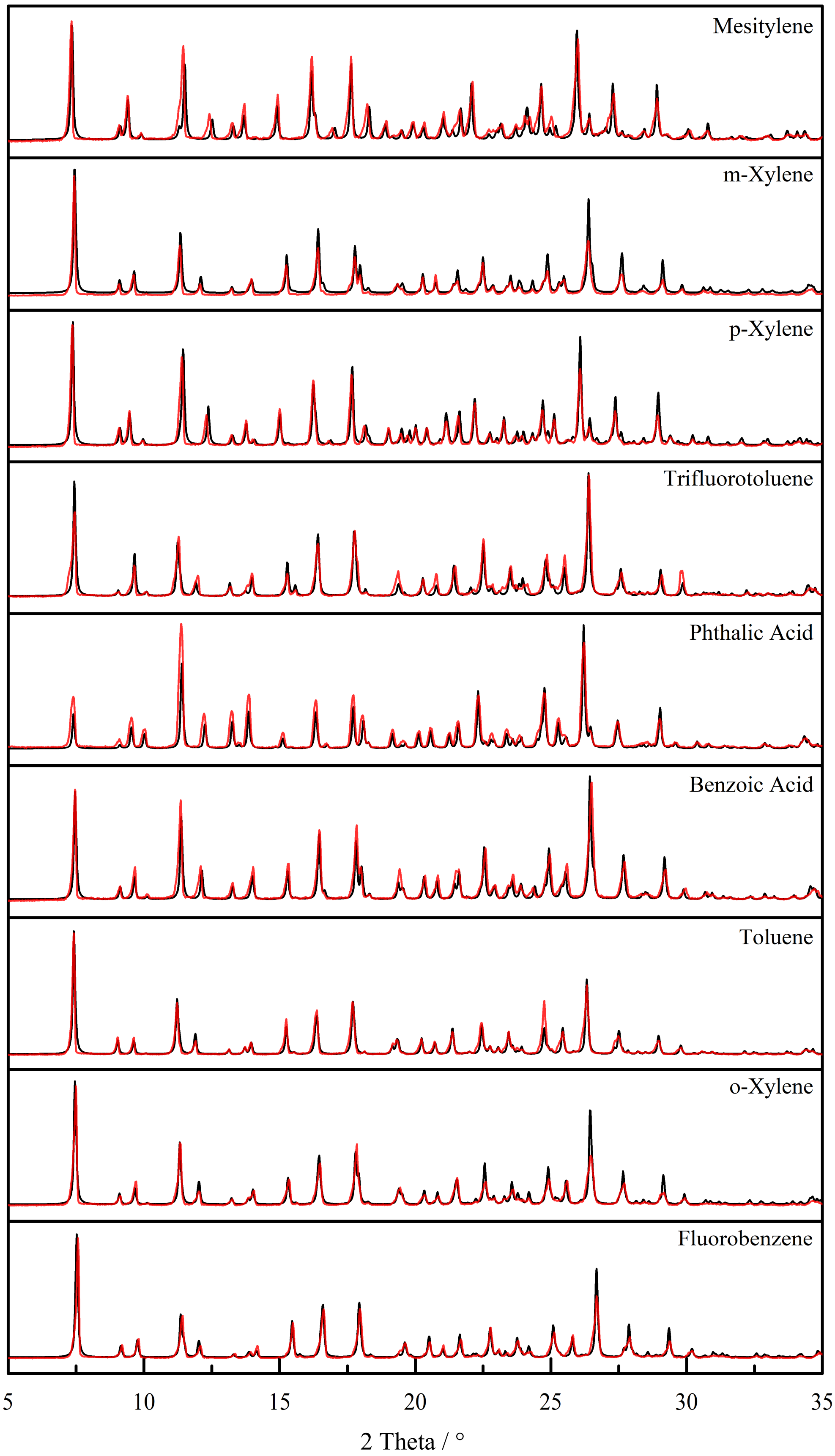

Fig. S1: Comparison of measured XRPD patterns (red line) and calculated XRPD patterns (black line) from crystal structure.

Note that SCXRD measurement was performed at either 120 K/95 K and the XRPD measurement of the prepared multicomponent forms was performed at room temperature. The patterns shown in Fig. S1 were corrected for equal temperature to simplify the comparison. Differences in peak intensity might be caused by preferential orientation of the crystals during XRPD measurements. Isostructurality of prepared samples is clearly visible from the XRPD patterns which are very similar.

DSC

DSC was measured to ensure solid phase purity of the prepared samples. Furthermore, melting temperatures of the multicomponent forms were used to correlate with other evaluated properties. Melting temperature of apremilast used in the original drug product is included as well. Evaluated melting temperatures are summarized in Fig. S2 and Tab. S2. The DSC diagrams used for the evaluation are shown in Fig. S3.

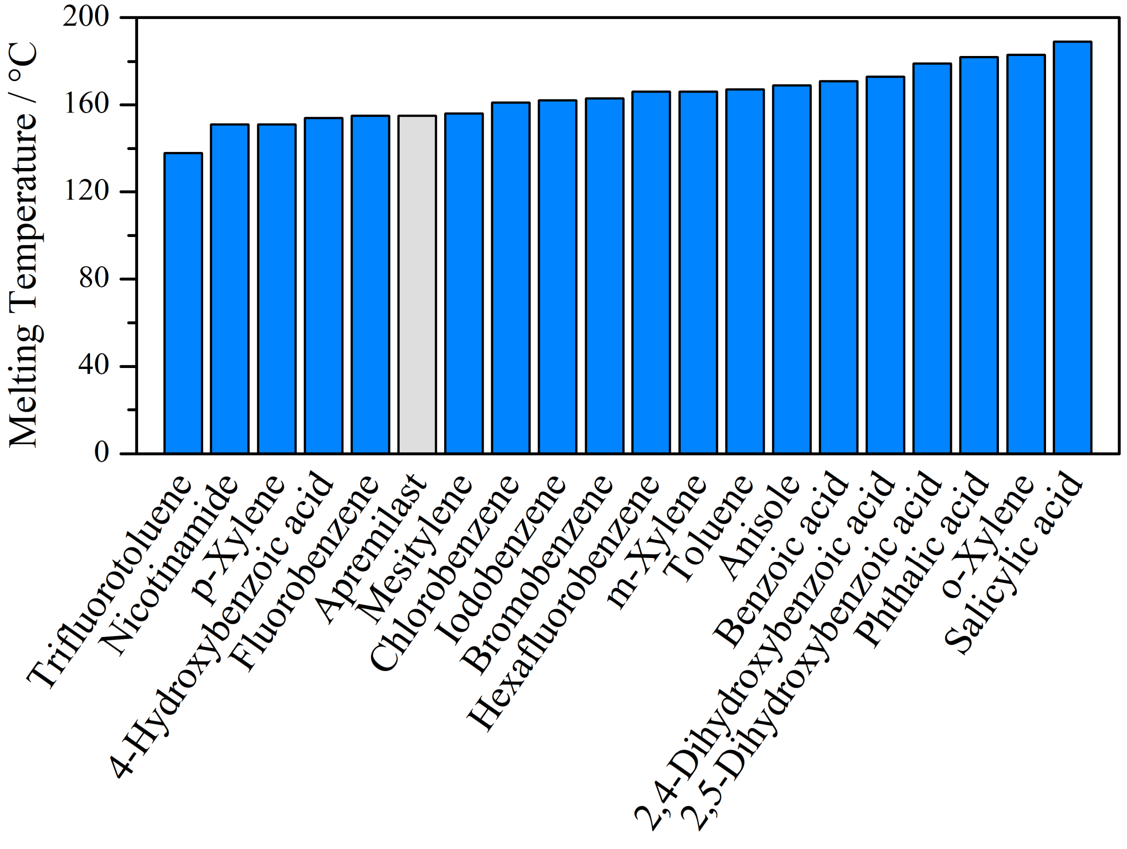

Fig. S2: Melting temperatures of all compared multicomponent forms and apremilast.

Tab. S3: Values of melting temperatures of all compared multicomponent forms and apremilast.

Multicomponent form	Melting temperature [°C]
Trifluorotoluene	138
Nicotinamide	151
p-Xylene	151
4-Hydroxybenzoic acid	154
Fluorobenzene	155

Apremilast	155
Mesitylene	156
Chlorobenzene	161
Iodobenzene	162
Bromobenzene	163
Hexafluorobenzene	166
m-Xylene	166
Toluene	167
Anisole	169
Benzoic acid	171
2,4-Dihydroxybenzoic acid	173
2,5-Dihydroxybenzoic acid	179
Phthalic acid	182
o-Xylene	183
Salicylic acid	189

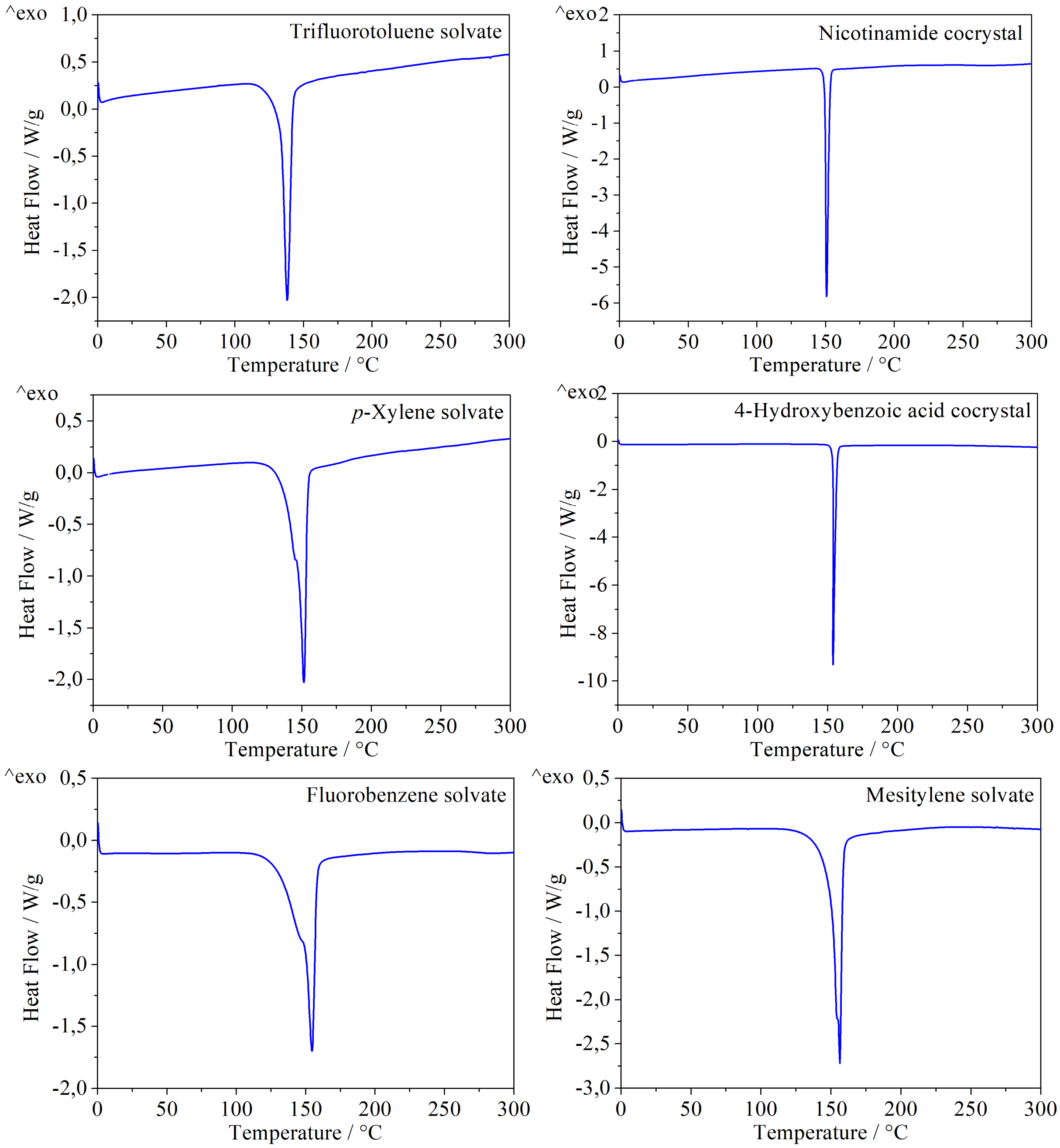
Trifluorotoluene solvate
Nicotinamide cocrystal
p-Xylene solvate
4-Hydroxybenzoic acid cocrystal
Fluorobenzene solvate
Mesitylene solvate
^exo
Heat Flow / W/g
Temperature / °C

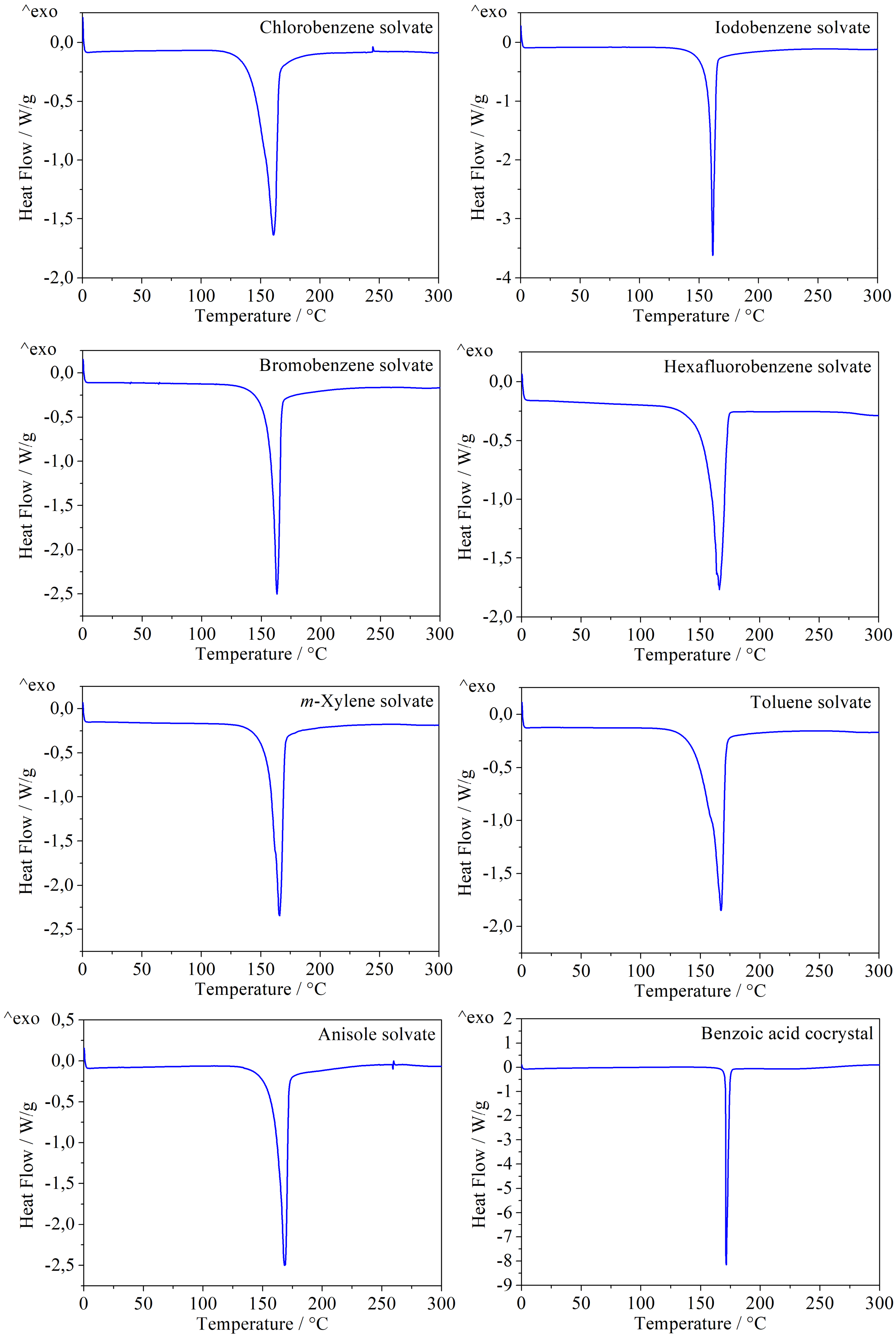
Chlorobenzene solvate
Iodobenzene solvate
Bromobenzene solvate
Hexafluorobenzene solvate
m-Xylene solvate
Toluene solvate
Anisole solvate
Benzoic acid cocrystal
^exo
Heat Flow / W/g
Temperature / °C

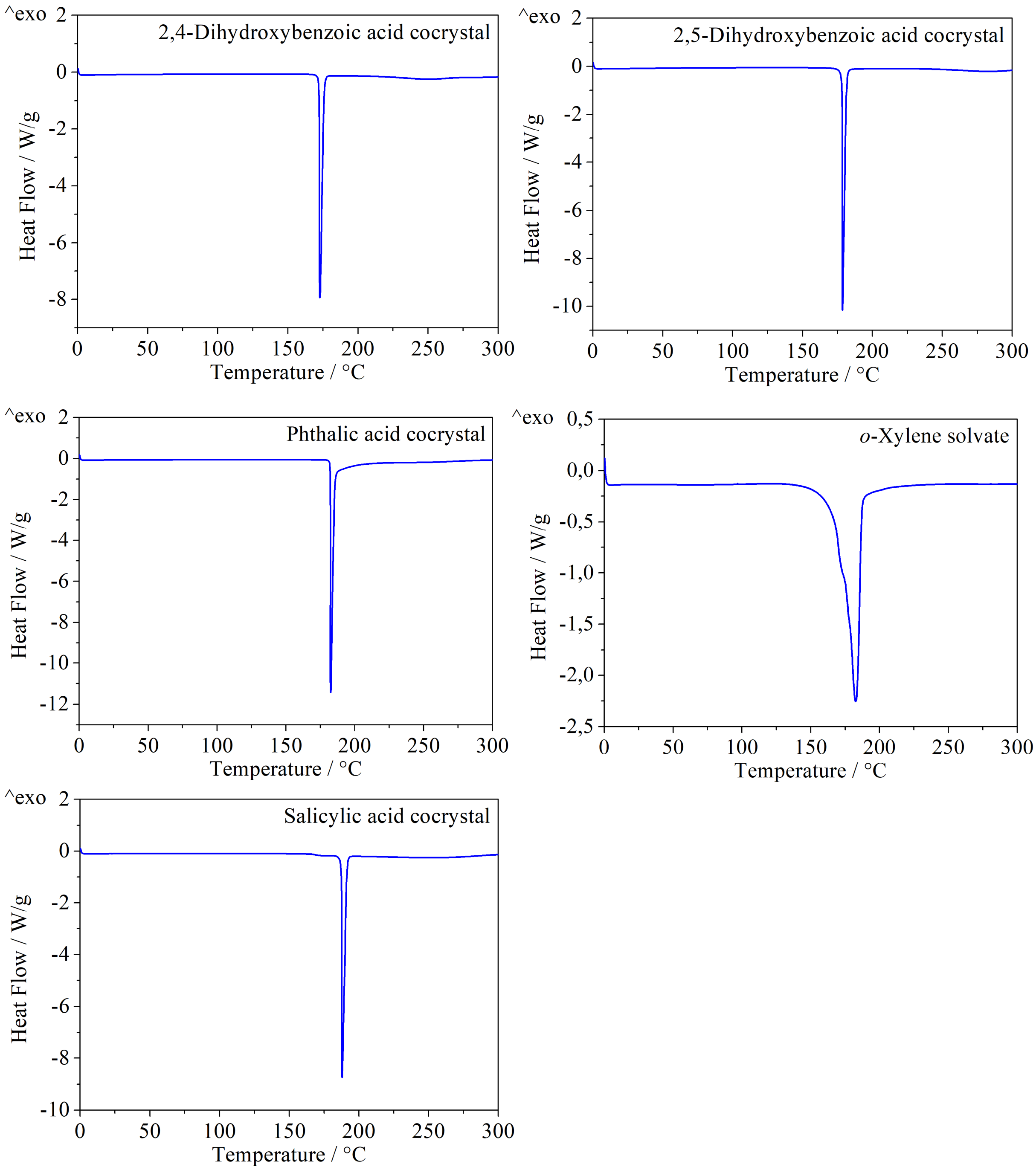

Fig. S3: DSC diagrams of all presented multicomponent forms.

Despite the melting temperatures of the guest molecules being publicly available information, the values used throughout this work are summarized in Tab. S4.

Tab. S4: Values of melting temperatures of all compared guest molecules.

Guest molecule	Melting temperature [°C]
Toluene	-95
m-Xylene	-48
Mesitylene	-45
Fluorobenzene	-44
Chlorobenzene	-44

Anisole	-37
Bromobenzene	-31
Iodobenzene	-29
Trifluorotoluene	-29
o-Xylene	-25
Hexafluorobenzene	5
p-Xylene	13
Benzoic acid	122
Nicotinamide	128
Salicylic acid	159
2,5-Dihydroxybenzoic acid	206
Phthalic acid	207
2,4-Dihydroxybenzoic acid	210
4-Hydroxybenzoic acid	215

IDR

Intrinsic dissolution rate of the measured forms was measured, evaluated, and correlated with other properties. It was also compared with the IDR of apremilast in the original drug product and it was observed that some of the new forms are able to bring substantial pharmaceutical advantages (however, not all of them could be used due to toxicity reasons). IDR values are summarized in Fig. S4 and Tab. S5.

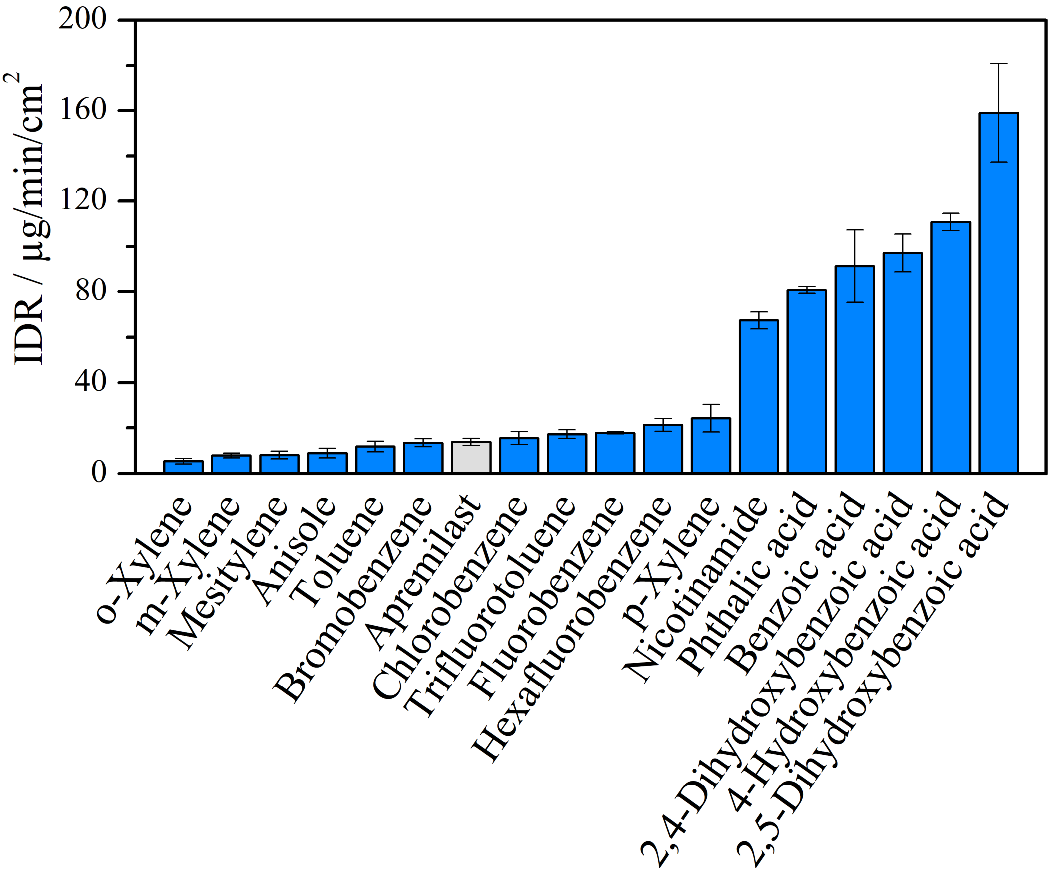

Fig. S4: Intrinsic dissolution rates of all compared multicomponent forms and apremilast.

Tab. S5: Values of intrinsic dissolution rates of all compared multicomponent forms and apremilast.

Multicomponent form	IDR [µg/min/cm^2]	Standard deviation
o-Xylene	5	1.2
m-Xylene	8	1.0
Mesitylene	8	1.6
Anisole	9	2.1
Toluene	12	2.3
Bromobenzene	13	1.7
Apremilast	14	1.5
Chlorobenzene	16	2.8
Trifluorotoluene	17	1.9
Fluorobenzene	18	0.6
Hexafluorobenzene	21	2.7
p-Xylene	24	6.1
Nicotinamide	67	3.7
Phthalic acid	81	1.5
Benzoic acid	91	15.9
2,4-Dihydroxybenzoic acid	97	8.3
4-Hydroxybenzoic acid	111	3.8
2,5-Dihydroxybenzoic acid	159	23.1

Measurement of iodobenzene solvate and salicylic acid cocrystal was repeated 10 times, but due to the disc breakage during the IDR measurement it was not possible to obtain usable data for those two solid forms. Therefore, those samples were not included in correlations between intrinsic dissolution rate and other measured quantities. It is interesting to note that IDR of cocrystals was in all cases higher compared to solvates.

EqSol

Equilibrium solubility of the prepared solid forms was measured in pH = 6.8 phosphate buffer with the addition of 0.2% of SDS. The tested solid form was mixed with the buffer and the formed slurry was stirred for 24 h at 750 RPM at room temperature. The solid and liquid phase were separated using centrifugal filtration (4000 RPM) and apremilast content in the liquid phase was analyzed using HPLC. EqSol values were used to correlate with other properties and are summarized in Fig. S5 and Tab. S6.

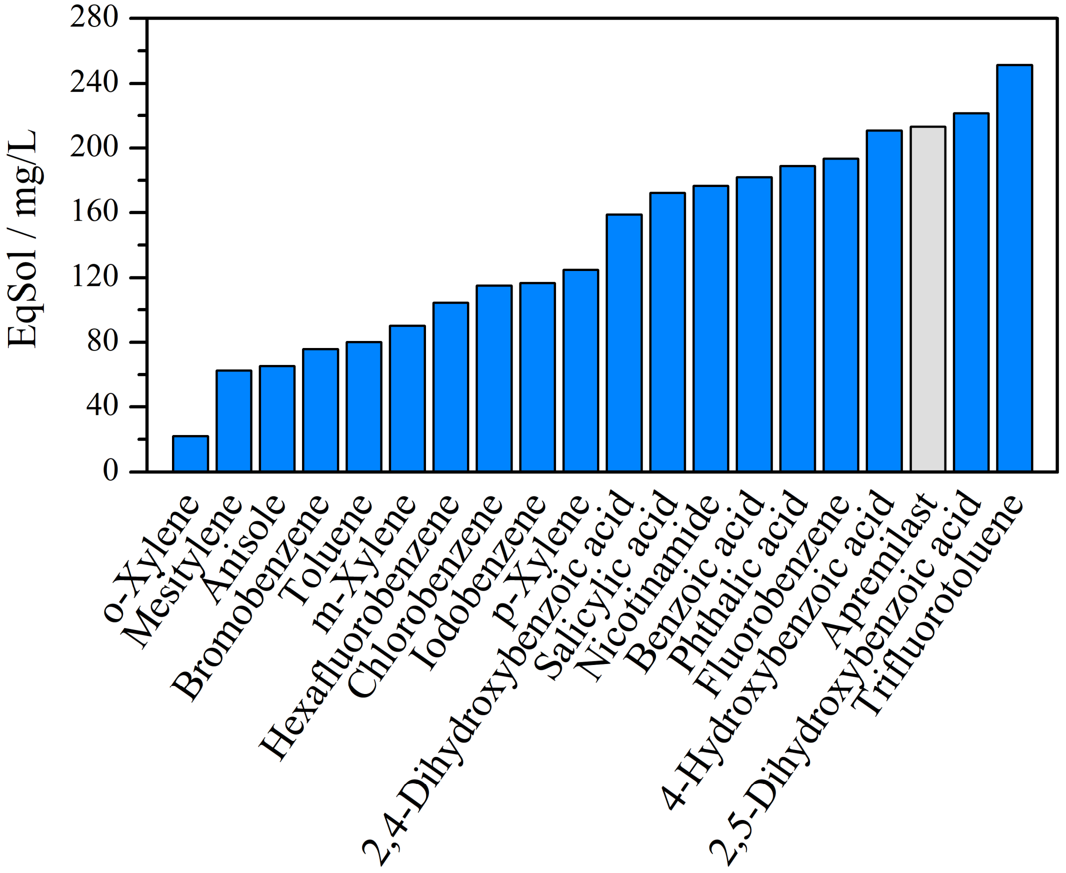

Fig. S5: Equilibrium solubility of all compared multicomponent forms and apremilast.

Tab. S6: Values of equilibrium solubility of all compared multicomponent forms and apremilast.

Multicomponent form	EqSol [mg/L]
o-Xylene	22
Mesitylene	63
Anisole	65

Bromobenzene	76
Toluene	80
m-Xylene	90
Hexafluorobenzene	104
Chlorobenzene	115
Iodobenzene	117
p-Xylene	125
2,4-Dihydroxybenzoic acid	159
Salicylic acid	172
Nicotinamide	177
Benzoic acid	182
Phthalic acid	189
Fluorobenzene	193
4-Hydroxybenzoic acid	211
Apremilast	213
2,5-Dihydroxybenzoic acid	222
Trifluorotoluene	251

Phase transformations of the solids during the experiment were considered and the powder samples used for the EqSol experiment were examined via Raman spectroscopy before and after the experiment (Fig. S6). It was observed that solvates are extremely stable over the duration of the experiment (24 hours in dissolution medium). No transformation was observed. Phase stability of cocrystals was varying from sample to sample exhibiting different levels of transformation to apremilast form II[9]. The lowest phase stability was estimated for 4-hydroxybenzoic acid cocrystal with approx. 50% conversion to apremilast form II after 24 h in the dissolution medium. The transformation suggests that there is no complex between the guest molecule and apremilast that would be stable enough to persist in the solution. It also implies that two forms contribute to the equilibrium solubility– the tested cocrystal and apremilast form II. It is interesting to note that conversion to form II appears mostly from multicomponent forms with higher equilibrium solubility as well as IDR (cocrystals).

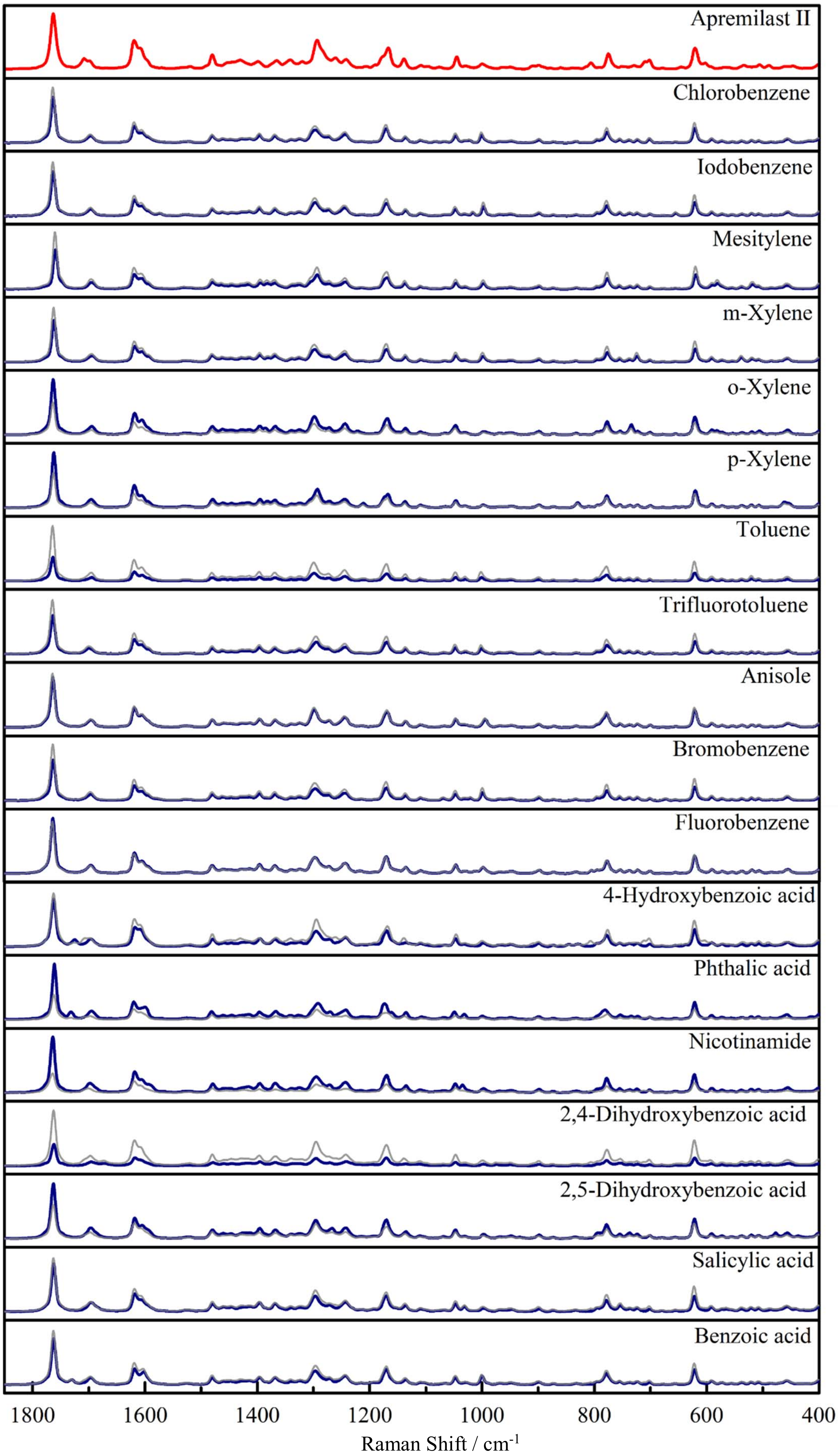
Apremilast II
Chlorobenzene
Iodobenzene
Mesitylene
m-Xylene
o-Xylene
p-Xylene
Toluene
Trifluorotoluene
Anisole
Bromobenzene
Fluorobenzene
4-Hydroxybenzoic acid
Phthalic acid
Nicotinamide
2,4-Dihydroxybenzoic acid
2,5-Dihydroxybenzoic acid
Salicylic acid
Benzoic acid
1800
1600
1400
1200
1000
800
600
400
Raman Shift / cm-1

Fig. S6: Comparison of Raman spectra before and after 24 hours of equilibrium solubility experiment in phosphate buffer dissolution medium. Blue line – before EqSol experiment. Grey line – after EqSol experiment.

Values for equilibrium solubility of guest molecules in water were find in scientific literature and are summarized in Fig. S7 and Tab. S7. References to scientific literature are also included in Fig. S7 and Tab. S7. Nicotinamide is not included in Fig. S7 to increase clarity, since its solubility in water is much higher compared to the rest of the guest molecules.

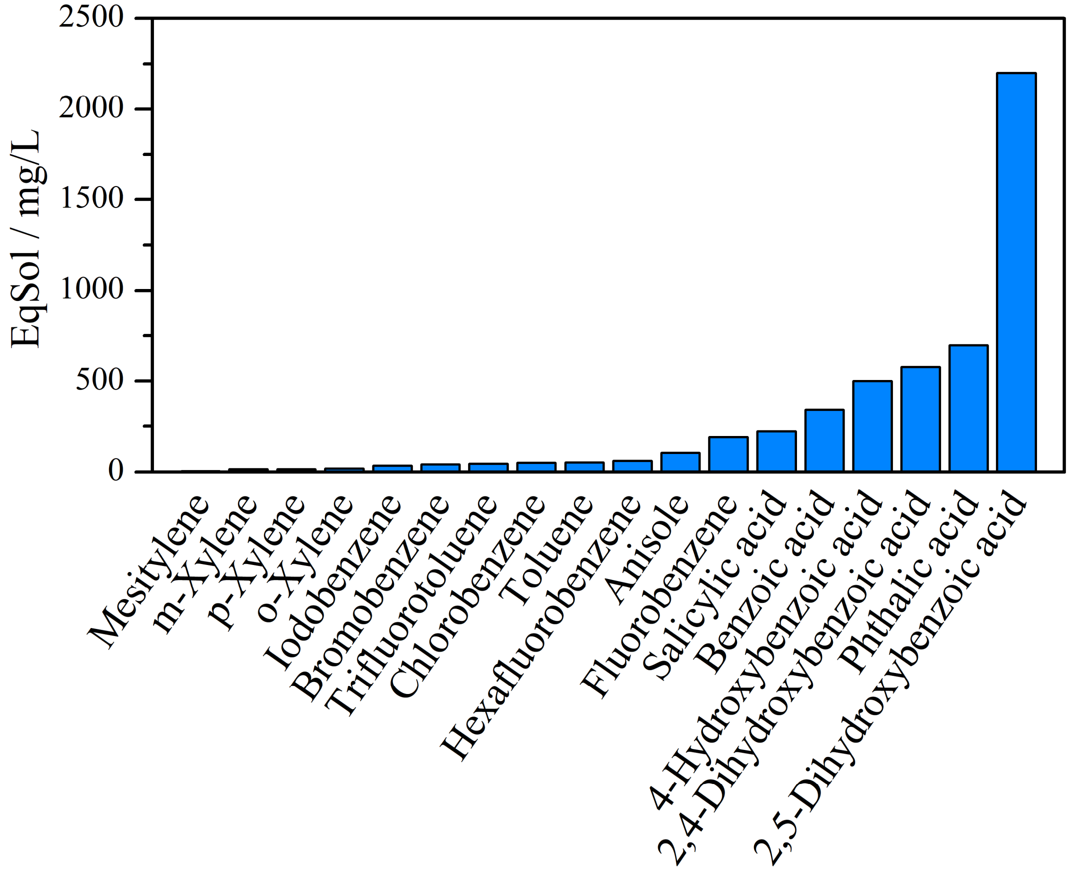

Fig. S7: Equilibrium solubility of all compared guest molecules. Nicotinamide is not shown to increase clarity.

Tab. S7: Values of equilibrium solubility of all compared guest molecules.

Guest molecule	Solubility [mg/L] + reference
Mesitylene	48 (ref [10])
m-Xylene	160 (ref [11])
p-Xylene	170 (ref [11])
o-Xylene	180 (ref[11])
Iodobenzene	340 (ref[12])
Bromobenzene	410 (ref [13])
Trifluorotoluene	450 (ref [14])
Chlorobenzene	500 (ref [11])
Toluene	530 (ref [15])

Hexafluorobenzene	617 (ref[16])
Anisole	1040 (ref [13])
Fluorobenzene	1920 (ref [12])
Salicylic acid	2240 (ref [13])
Benzoic acid	3400 (ref [11])
4-Hydroxybenzoic acid	5000 (ref [17])
2,4-Dihydroxybenzoic acid	5780 (ref [18])
Phthalic acid	6970 (ref [10])
2,5-Dihydroxybenzoic acid	22000 (ref [19])
Nicotinamide	1000000 (ref [20])

All solubility values of guest molecules, as well as references (Tab. S7), were taken from database PubChem (experimental values were used). Nicotinamide was not included into correlations of guest molecule solubility with other properties because its solubility is several orders of magnitude higher compared to the rest of the guest molecules.

CrystalCMP

Crystal CMP was used to compare packing similarity of solved structures. Since many readers might not be familiar with the software used for this purpose, there is a short description of the CrystalCMP software. The CrystalCMP software works differently compared to for example Crystal Packing Similarity tool used in Mercury (one of the tools many readers might be familiar with). The comparison of the clusters within Mercury is based on the calculations of the differences between the interatomic positions and the similarity/difference is calculated as positional difference between molecules in a molecular cluster. CrystalCMP on the other hand, calculates the packing similarity differently. The similarity is calculated from the differences between positions and rotations between the center of the related molecules in the cluster. The deviations of the positions and rotations are considered in the packing similarity term (PS) as follows:

$$PS = D + X \cdot \frac{A}{180},$$

where D is the average distance (in Å) between molecular centers of related molecules and A is the average angle between them. X is a default parameter that weighs the influence of the A parameter (is set to 100 by default). The values of PS are plotted on the x-axis for the different solid forms showing the differences in the packing. The compared structures are more similar in terms of crystal packing the smaller the difference in PS is. For the calculations is used only the biggest molecule of the cluster (apremilast in this case), thus ignoring the guest molecule. For more details refer to the original publication[21].

Bibliography

1. FDA approval letter. https://www.accessdata.fda.gov/drugsatfda_docs/nda/2014/206088Orig1s000Approv.pdf (accessed 03.04.2020).
2. Palatinus, L.; Chapuis, G., SUPERFLIP - a computer program for the solution of crystal structures by charge flipping in arbitrary dimensions. *J. Appl. Crystallogr.* **2007,** *40* (4), 786-790.
3. Betteridge, P. W.; Carruthers, J. R.; Cooper, R. I.; Prout, K.; Watkin, D. J., CRYSTALS version 12: software for guided crystal structure analysis. *J. Appl. Crystallogr.* **2003,** *36* (6), 1487.
4. Rohlicek, J.; Husak, M., MCE2005 - a new version of a program for fast interactive visualization of electron and similar density maps optimized for small molecules. *J. Appl. Crystallogr.* **2007,** *40* (3), 600-601.
5. Dudek, M. K.; Kostrzewa, M.; Paluch, P.; Potrzebowski, M. J., pi-Philic Molecular Recognition in the Solid State as a Driving Force for Mechanochemical Formation of Apremilast Solvates and Cocrystals. *Cryst. Growth Des.* **2018,** *18* (7), 3959-3970.
6. Wang, F. Y.; Zhang, Q.; Zhang, Z. Y.; Gong, X. Y.; Wang, J. R.; Mei, X. F., Solid-state characterization and solubility enhancement of apremilast drug-drug cocrystals. *CrystEngComm* **2018,** *20* (39), 5945-5948.
7. Wu, Y. D.; Zhang, X. L.; Liu, X. H.; Xu, J.; Zhang, M.; Shen, K.; Zhang, S. H.; He, Y. M.; Ma, Y.; Zhang, A. H., The preparation, characterization, structure and dissolution analysis of apremilast solvatomorphs. *Acta Crystallogr., Sect. C: Struct. Chem.* **2017,** *73*.
8. Jirát, J.; Zvoníček, V.; Babor, M.; Ridvan, L.; Skořepová, E.; Dušek, M.; Šoóš, M., Formation of the First Non-Isostructural Cocrystal of Apremilast Explained. *Cryst. Growth Des.* **2020**.
9. Nian, J. Stable Apremilast crystal form II free of solvates, and preparation method therefor. WO2016141503A1, 2016.
10. Yalkowsky, *Handbook of Aqueous Solubility Data: An Extensive Compilation of Aqueous Solubility Data for Organic Compounds Extracted from the AQUASOL dATAbASE*. CRC Press LLC: 2003.
11. Yalkowsky, *Handbook of Aqueous Solubility Data Second Edition*. CRC Press: 2010.
12. Chiou, C. T.; Freed, V. H.; Schmedding, D. W.; Kohnert, R. L., Partition coefficient and bioaccumulation of selected organic chemicals. *Environmental Science & Technology* **1977,** *11* (5), 475-478.
13. Yalkowsky, *AQUASOL Database of Aqueous Solubility*. University of Arizona: 1992.
14. Valvani, S. C.; Yalkowsky, S. H.; Roseman, T. J., Solubility and partitioning IV: Aqueous solubility and octanol-water partition coefficients of liquid nonelectrolytes. *J. Pharm. Sci.* **1981,** *70* (5), 502-7.
15. Isao, S.; Masatake, A.; Toshio, D.; Hideo, N., Solubility Measurements of Benzene and the Alkylbenzenes in Water by Making Use of Solute Vapor. *Bull. Chem. Soc. Jpn.* **1982,** *55* (4), 1054-1062.
16. Freire, M. G.; Razzouk, A.; Mokbel, I.; Jose, J.; Marrucho, I. M.; Coutinho, J. A. P., Solubility of Hexafluorobenzene in Aqueous Salt Solutions from (280 to 340) K. *J. Chem. Eng. Data* **2005,** *50* (1), 237-242.
17. Yalkowsky, *Handbook of Aqueous Solubility Data: An Extensive Compilation of Aqueous Solubility Data for Organic Compounds Extracted from the AQUASOL dATAbASE*. CRC Press LLC: 2003.
18. BEILSTEIN https://pubchem.ncbi.nlm.nih.gov/compound/2_4-Dihydroxybenzoic-acid.
19. Yalkowsky, *Handbook of aqueous solubility data*. CRC Press: 2003.
20. Niacin, Nicotinamide, and Nicotinic Acid. In *Kirk-Othmer Encyclopedia of Chemical Technology*.
21. Rohlicek, J.; Skorepova, E.; Babor, M.; Cejka, J., CrystalCMP: an easy-to-use tool for fast comparison of molecular packing. *J. Appl. Crystallogr.* **2016,** *49* (6), 2172-2183.
